# A New C-3/C-3”-Biflavanone from the Roots of *Stellera chamaejasme* L

**DOI:** 10.3390/molecules16086465

**Published:** 2011-07-29

**Authors:** Jie Li, Wei Zhao, Jia-Lei Hu, Xin Cao, Jie Yang, Xiang-Rong Li

**Affiliations:** School of Medicine and Life Sciences, Zhejiang University City College, No. 48, Huzhou Road, Hangzhou 310015, Zhejiang, China

**Keywords:** *Stellera chamaejasme*, Thymelaeaceae, biflavanone

## Abstract

A new 3, 3”-biflavanone, neochamaejasmin C (**1**), was isolated from the roots of *Stellera chamaejasme* L., together with four known compounds. Their structures and configurations were elucidated by spectroscopic methods, including 2D-NMR techniques.

## 1. Introduction

**Figure 1:**
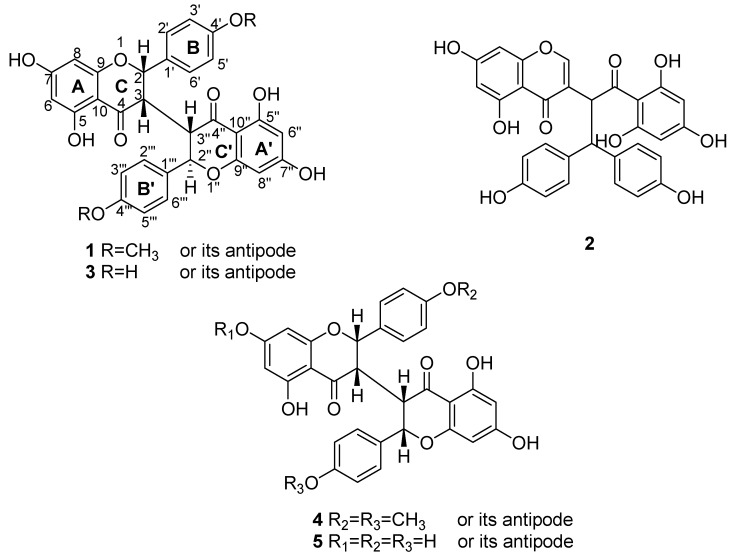
Chemical Structures of **1-6**.

The plant *Stellera chamaejasme* L. (Thymelaeaceae) is a well-recognized traditional Chinese herbal medicine, widely distributed in the north and southwest of China. Its roots are commonly used for the treatment of scabies, tinea, stubborn skin ulcers, chronic tracheitis, and tuberculosis [1]. Previous studies on the chemical constituents of this plant have resulted in reports on the presence of groups of biflavonoids, lignans, diterpenes, *etc.* [2,3,4,5,6,7,8,9]. To achieve a deeper understanding of the chemistry of this crude drug, further phytochemical research on this plant was carried out by our group. One new 3,3”-biflavanone, neochamaejasmin C (**1**, Figure 1), together with four known compounds, namely chamaechromone (**2**), neochamaejasmin B (**3**), chamaejasmenin B (**4**), and chamaejasmenin C (**5**) (Figure 1) were isolated from the roots of *Stellera chamaejasme* L. Details of the isolation and structure elucidation of the compound **1** are presented herein.

## 2. Results and Discussion

**Figure 2:**
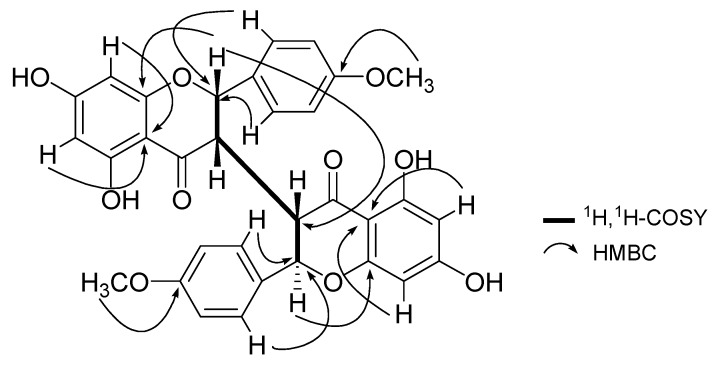
key ^1^H-^1^H COSY and HMBC correlations of **1**.

Neochamaejasmin C (**1**) was obtained as an amorphous powder. Its molecular formula C_32_H_26_O_10_was established by HR-ESI-MS (m/z 569.1446 [M-H]^-^). The color development reaction with HCl-Mg reagent showed a red color, indicating that **1** is a flavonoid. The ^1^H-NMR spectrum of **1** (Table 1) displayed signals of two methoxyl groups (*δ*_H_ 3.79, 3.82, s, each 3H), two protons of H-2 (*δ*_H_ 5.77, 1H, d, *J* = 4.8 Hz), H-2” (*δ*_H_ 5.22, 1H, d, *J* = 8.8 Hz), and two protons of H-3 (*δ*_H_ 3.24, 1H, br s), H-3” (*δ*_H_ 4.03, 1H, dd, *J* = 8.8, 3.4 Hz). The aromatic proton signals (*δ*_H_ 5.86-7.36, 12H) indicated the presence of two sets of typical 5,7-dioxygenated A rings (*δ*_H_ 5.95, 6.08, each 1H, d, *J* = 2.0 Hz; *δ*_H_ 5.87, 5.86, each 1H, d, *J* = 2.4 Hz), and two sets of *para*-oxygenated B rings (*δ*_H_ 7.36, 6.98, each 2H, d, *J* = 8.6 Hz; *δ*_H_ 7.10, 6.84, each 2H, d, *J* = 8.6 Hz). From the ^13^C-NMR data (Table 1), two carbonyl (*δ*_C_ 195.0, 197.2) and two methoxyl groups (*δ*_C_ 54.8) were also observed. On the basis of these observations, the structure of compound **1** was determined to consist of two flavanone units [2,3,4,5,6,7]. The partial (-CH-CH-CH-CH-) structure inferred from the ^1^H-^1^H COSY spectrum (bold line in Figure 2) suggested that the linkage of the two flavanones was possible only at the C-3 and C-3” positions, which was supported by the comparison of the ^1^H- and ^13^C-NMR data of **1** with those known 3,3”-biflavanones [2,3,4,5,6,7,8,9], and further confirmed by the HMBC correlations of H-2 (*δ*_H_ 5.77) with C-3”(*δ*_C_ 49.3). The linkage of the B ring to the C ring was established at C-2 by HMBC (Figure 2) experiment, in which H-2’ and H-6’ (*δ*_H_ 7.36) correlated with C-2 (*δ*_C_ 79.9). In the same way, linkage of the B’ ring to C-2” of the C’ ring was deduced by the correlations of H-2”’ and H-6”’ (*δ*_H_ 7.10) with C-2” (*δ*_C_ 81.8). The HMBC cross-peaks between two methoxyl groups with C-4’ and C-4”’ on B and B’ rings indicated that two methoxyl groups were connected to C-4’ and C-4”’ respectively.

**Table 1:** NMR data of compound **1** in acetone-*d_6_* (500 MHz for ^1^H, 125 MHz for ^13^C).

No.	*δ*_H_ mult (*J* = Hz)	*δ*_C_
2	5.77 d (4.8)	79.9 d
3	3.24 br s	48.0 d
4	-	197.2 s
5	-	164.2 s
6	5.95 d (2.0)	96.2 d
7	-	166.5 s
8	6.08 d (2.0)	95.3 d
9	-	163.9 s
10	-	104.0 s
1’	-	128.7 s
2’	7.36 d (8.6)	127.4 d
3’	6.98 d (8.6)	114.1 d
4’	-	159.8 s
5’	6.98 d (8.6)	114.1 d
6’	7.36 d (8.6)	127.4 d
2”	5.22 d (8.8)	81.8 d
3”	4.03 dd (8.8, 3.4)	49.3 d
4”	-	195.0 s
5”	-	164.5 s
6”	5.87 d (2.4)	96.0 d
7”	-	166.7 s
8”	5.86 d (2.4)	94.9 d
9”	-	162.1 s
10”	-	102.8 s
1”’	-	128.8 s
2”’	7.10 d (8.6)	129.1 d
3”’	6.84 d (8.6)	113.8 d
4”’	-	160.1 s
5”’	6.84 d (8.6)	113.8 d
6”’	7.10 d (8.6)	129.1 d
4’-OCH_3_	3.82 s	54.8 q
4’”-OCH_3_	3.79 s	54.8 q

The stereochemistry at the C-2/C-3 and C-2”/C-3” positions in **1** were determined as *cis-trans* geometry by comparison of the *J* values (*J*_H-2_ = 4.8 Hz and *J*_H-2”_ = 8.8 Hz) with those of the known 3,3”-biflavanones. The relative configuration of **1** was assigned to be identical to that of neochamaejasmin B (**3**) due to the similarity of their chemical shifts and coupling constants of all H-atoms on H-2, H-3, H-2”, and H-3”, the key NOESY correlations between H-2” (*δ*_H_ 5.22) with H-2’(H-6’) (*δ*_H _7.36) further confirm the conclusion above. Thus, on the basis of the above evidence, the structure of **1** was established to be 5,5’,7,7’-tetrahydroxy-2-(4-methoxyphenyl)-2’-(4-methoxy-phenyl) [3,3’-bi-2*H*-1-benzopyran]-4,4’(3*H*,3’*H*)-dione, and named neochamaejasmin C. The known compounds were identified as chamaechromone (**2**) [4], neochamaejasmin B (**3**) [3], chamaejasmenin B (**4**) [6], and chamaejasmenin C (**5**) [6] by comparison of their ^1^H- and ^13^C-NMR and MS data with published data.

## 3. Experimental

### 3.1. General

Melting points were measured on a Thermal Values analytical microscope and were uncorrected. Optical rotations were recorded on a Perkin-Elmer 341 polarimeter. IR spectra were recorded on a Nicolet FI-IR 200SXY spectrophotomer. ^1^H- and ^13^C-NMR spectra were measured in (D_6_) acetone with TMS as the internal standard on a Bruker DMX-500 NMR instrument. Silica gel G_254_ and H (Qingdao Sea Chemical Factory, China) were used for TLC and column chromatography, respectively.

### 3.2. Plant Material

The roots of *Stellera chamaejasme* L. were collected in Kunming, Yunnan Province, China, in October, 2010. The plant was identified by Le Cai (Yunnan University). A voucher specimen was deposited with the Zhejiang University City College.

### 3.3. Extraction and Isolation

Air-dried powdered roots (3.0 kg) of *S. chamaejasme* were extracted exhaustively with 95% aq. EtOH (5 L) at r. t. for four times. After concentration *in vacuo*, a crude extract (360 g) was obtained, which was suspended in 1 L H_2_O, and the suspension was extracted three times successively with petroleum ether (PE) (1 L), AcOEt (1 L), and BuOH (1 L) at r. t. to yield 45, 160 and 86 g of each extract, respectively. The EtOAc extract was subjected to column chromatography (CC) with PE/AcOEt gradient system of increasing polarity (9:1, 8:2, 7:3, 6:4, 5:5) to give six fractions (Fr. 1-6). Fraction 3 was chromatographed repeatedly over a SiO_2_ column with MeOH/H_2_O (7:3, 8:2, 9:1) to afford **5** (18 mg), **4** (30 mg), and **1** (24 mg). Fraction 7 was rechromatographed on a SiO_2_ column with CHCl_3_/MeOH (99:1, 98:2, 94:6) to give **3** (16 mg). Fraction 10 was chromatographed over a SiO_2 _column with CHCl_3_/MeOH (99:1, 98:2, 95:5) to yield **2** (170 mg).

*Neochamaejasmin C* (**1**). Yellow amorphous powder, mp 216–218 °C; [α]+226 (c=0.65, MeOH); IR (KBr) cm^−1^: 3382, 1640; ^1^H-NMR and ^13^C-NMR data, see Table 1; HR-ESI-MS: m/z 569.1446 [M-H]^−^, calcd for C_32_H_25_O_10_, 569.1448.

## 4. Conclusions

In conclusion, one new biflavanone, 5,5’,7,7’-tetrahydroxy-2-(4-methoxyphenyl)-2’-(4-methoxy-phenyl) [3,3’-bi-2*H*-1-benzopyran]-4,4’(3*H*,3’*H*)-dione (**1**), together with four known compounds, chamaechromone (**2**), neochamaejasmin B (**3**), chamaejasmenin B (**4**), and chamaejasmenin C (**5**), were isolated from the EtOH extract of the roots of *Stellera chamaejasme* L.
